# Effects of self-management, education and specific exercises, delivered by health professionals, in patients with osteoarthritis of the knee

**DOI:** 10.1186/1471-2474-9-133

**Published:** 2008-10-02

**Authors:** S Coleman, NK Briffa, G Carroll, C Inderjeeth, N Cook, J McQuade

**Affiliations:** 1 Arthritis Foundation of Western Australia, PO Box 34, Wembley, Western Australia, 6914, Australia; 2 Department of Physiotherapy, Curtin University of Technology, Bentley, Western Australia, 6102, Australia; 3 ArthroCare Pty Ltd, Department of Rheumatology, Fremantle Hospital, University of Notre Dame Australia, Western Australia, 6055, Australia; 4 Gerontology, Sir Charles Gairdner Hospital, Verdun St, Nedlands, Western Australia, 6009, Australia; 5 Rheumatology, Royal Perth Hospital, Wellington St, Perth, Western Australia, 6001, Australia

## Abstract

**Background:**

An education self-management program for people with osteoarthritis (OA) of the knee was designed to be delivered by health professionals, incorporating their knowledge and expertise. Improvement in quality of life, health status and pain in response to this program has previously been demonstrated in an uncontrolled pilot study. To more rigorously test the effectiveness of the program we will undertake a randomised controlled trial of people with OA of the knee offering specific self-administered exercises and education, in accordance with the principles of self-management.

Aim: To determine whether an education self management program for subjects with Osteoarthritis (OA) of the knee (OAK program) implemented by health professionals in a primary health care setting can achieve and maintain clinically meaningful improvements compared standard medical management in a control group.

**Methods:**

The effects of standard medical management will be compared with the effects of the OAK program in a single-blind randomized study.

*Participants: *146 male and female participants with established OA knee will be recruited. Volunteers with coexistent inflammatory joint disease or serious co-morbidities will be excluded.

*Interventions: *Participants will be randomized into either intervention or control groups (delayed start). The intervention group will complete the OA knee program and both groups will be followed for 6 months.

*Measurements: *Assessments will be at baseline, 8 weeks and 6 months. SF-36, WOMAC and VAS pain questionnaires will be completed. Isometric quadriceps and hamstring strength will be measured using a dynamometer; knee range of movement using a goniometer; and physical function will be determined by a modified timed up and go test. Data will be analysed using repeated measures ANOVA.

**Discussion:**

While there is evidence to support the effectiveness of SM programs for people with hypertension, diabetes and asthma, the evidence available for treatment of arthritis remains equivocal. The aim of this study is to determine the effectiveness of a disease specific self-management program for people with OA knee.

The study design includes all the important features of a clinical experimental study to minimize bias so the results of the study will provide a high level of evidence. People with OA of the knee have identified pain and problems with daily activities as the most important problems associated with their condition. The outcome measures selected specifically address these issues and have demonstrated validity and are responsive within the range of change expected in response to the intervention. Hence the results of the study will reflect their priorities.

The results of the study will provide evidence to guide clinicians and funding bodies seeking to establish priorities regarding the provision of this disease specific program.

**Trial registration:**

ACTR number: 12607000080426

## Background

Self-management is a primary care intervention that has become a popular component of management in a number of chronic conditions including arthritis. Unlike traditional patient education programs, self-management programs aim to achieve more than the provision of information to increase knowledge. They also aim to change health behavior and health status, teaching patients to identify and solve problems, set goals and plan actions [1].

Numerous self-management programs have been developed for different health conditions. Various models have been employed including individual and group-based programs that may be disease specific or generic [2]. Face-to-face interaction with health professionals is an important component of some programs, whereas trained lay leaders, usually presenting scripted information, deliver others.

There is a considerable body of research evaluating self-management programs. Reviews and meta-analyses [3,4] have shown that patient self-management education programs can significantly improve knowledge, compliance behaviours, and health outcomes, however the effectiveness differs between programs and disease states.

One systematic review of self-management interventions for a number of chronic diseases, found a trend towards a small benefit from arthritis programs, but the results were not significant and there was a suggestion of publication bias [2]. Many of the existing arthritis self-management programs are designed to cater for participants with any form of arthritis. Examples of this approach are the Chronic Diseases and Arthritis Self-Management programs (ASMP) developed at Stanford University [5,6]. These programs are also delivered in a group setting but they are led by trained lay tutors. They have a more generic approach as they are catering for participants with a variety of different musculoskeletal diseases in the one group. This approach may be cheaper to deliver but cost-effectiveness is yet to be established [7,8].

The ASMP has been tested widely with the majority of studies conducted in the USA or UK. Many, but not all of these studies have found the program to be effective. Overall, Warsi et al (2004), in their systematic review of self-management interventions for various chronic diseases, found a trend towards a small benefit from arthritis programs, the majority being ASMP or ASMP derivatives, but the results were not significant and here too it was suggested that there was publication bias [2].

We hypothesised that a program designed for a specific diagnostic group may be more effective. We considered a program of this nature would be justified for more prevalent conditions such as osteoarthritis of the knee.

Accordingly, we developed a self-management program for people with osteoarthritis of the knee. The program was designed to be delivered by health professionals. Strategies for pain management; the benefits of different types of exercise (strength, aerobic, flexibility, balance); and approaches for falls prevention were included. In keeping with a social cognitive theory approach, individual problem identification, goal setting and action planning were encouraged to facilitate improvements in self-efficacy. This program was tested in an uncontrolled quality assurance study. The results indicated improvement in pain, quality of life and physical function [9]. The purpose this study was to more rigorously test the program in a randomized controlled study.

## Methods

### Aim

To compare the effectiveness of an osteoarthritis of the knee self-management education program delivered by health professionals with a control group, as determined by improvements in pain, quality of life and physical function.

### Study Design

A two group randomized, controlled repeated measures study design will compare the osteoarthritis of the knee program (OAK) with a similar group of control participants. Independently of the study all participants will be able to receive standard medical management of OA knee. In addition, the intervention group will participate in a disease specific knee osteoarthritis self-management program (OAK). Blinding of participants will not be possible due to the nature of the intervention; however, assessors will be blinded to group allocation. The OAK program will be conducted in a community setting at Arthritis Western Australia.

### Hypothesis

People with osteoarthritis of the knee who complete the OAK Program will report improved quality of life, improved knee function and decreased pain compared with those managed conventionally.

### Ethical Issues

This study has been approved by the Human Research Ethics Committee at Curtin University of Technology (HR141, 2002). All participants will provide written informed consent prior to randomisation. Data access and storage will be in keeping with National Health and Medical Research Council guidelines, as approved. License agreements have been obtained for SF-36 Questionnaire and WOMAC Questionnaire. This trial is registered with the Australian and New Zealand Clinical Trial Registry, number: 12607000080426.

### Subjects

146 participants with established OA of one or both knees will be recruited into the study. As the OAK program is generally provided as a clinical service, subjects will be recruited from among people presenting to enroll in the OAK program. The operational definition for OA knee is diagnosis by a medical practitioner based on either clinical examination or radiological evidence. Disease severity is not a selection criterion. Exclusion criteria will be rheumatoid arthritis or other inflammatory joint disease; knee surgery planned within 6 months of commencing the study; physical impairments that preclude full participation; inability to communicate in English within a group setting or aged ≤ 18 years (Table 1). During the recruitment phase the program will be actively promoted to general practitioners and rheumatologists through professional societies, and to the general public through advertising and media coverage.

**Table 1:** Eligibility criteria

**Inclusion criteria**	**Exclusion criteria**
English speaking	Co-existing inflammatory arthritis
Aged 18 years or over	Serious co-morbidity
Diagnosis of OA (X-Ray or clinical Dx)	Scheduled knee replacement in < 6 months
Referral from GP or Specialist	Cannot meet program time-points
Able to meet program requirements	

Volunteers for the study will be randomized to an intervention group (immediate start) or a control group (delayed start). As there is evidence that SM is an effective addition to usual care [5,10], all volunteers randomized to the control group will be offered the intervention at the completion of the 6-month control period.

### Group allocation

Volunteers will be randomized in blocks to ensure manageable numbers for intervention groups. Pre-prepared cards indicating group assignment will be placed in sealed opaque envelopes and drawn as a lottery by a third party for allocation to treatment groups. In order to ensure optimum group sizes, allocation will not take place until a whole block has been recruited

### Intervention

The OAK program will be conducted over a 6-week period enabling participants to incorporate and consolidate information learned from week to week. In addition to the weekly sessions, participants will be given printed information relevant to the course component discussed each week. Program leaders will be health professionals including nurses, physiotherapists, and occupational therapists that have the knowledge and skills to present information on disease specific topics and accurately respond to complex questions. It is necessary that health professionals who deliver this program meet minimum musculoskeletal knowledge requirements. The fidelity of the OAK program will be maintained by the use of a facilitator's manual with modules for program delivery each week.

To facilitate optimum group dynamics, the target group size will be 12 participants, although this may vary from 8 to 16 depending on recruitment and randomisation. The program approach is holistic and will not exclusively focus on one aspect of care. Self-management constructs will be employed to promote behavioral changes that will be aimed at optimizing participants' health status. Goal setting and the development of strategies to achieve these goals long term [11] will be emphasized in the program. Participants will be encouraged to set their own goals related to health areas that they identify as requiring improvement.

Topics that will be covered in the weekly sessions include:

• Pain management strategies (cognitive and pharmaceutical)

• Joint protection

• Fitness/exercise

• Correct use of analgesia/medications

• Balance/falls prevention/proprioception

• Cognitive techniques

• Pathophysiology

• Nutrition/weight control

• Self-management skills

• Team approach to health care

• SMART goals (**S**pecific, **M**easurable, **A**chievable, **R**ealistic, **T**ime-framed)

### Assessments and Procedures

Assessments will be performed by 2 physiotherapists blinded to group allocation; conducted one week prior to the first self-management session (baseline) and on the week following the sixth and final session (week 8). The physiotherapists performing assessments will have no contact with the participants other than during the assessment sessions and will not participate in facilitation of the program. Participants will be assessed by the same physiotherapist whenever possible to ensure consistency.

Members of the control group will also attend assessments at baseline, and week 8. All volunteers will be assessed again 6 months after randomisation. In keeping with intention to treat principles, all participants will be encouraged to attend for follow-up measurements regardless of level of attendance at the self-management intervention. Self reported questionnaires (WOMAC, SF-36, VAS pain) would be mailed out to participants that are unable to attend assessment visits. Program and assessment attendance (at all time-points) will also be collected and collated.

Demographic information will include age, sex, co-morbidities and socioeconomic information. Socio-economic scales will be compiled using "The Index of Relative Socio-Economic Disadvantage" scales [12]. This index provides a weighted value that includes variables that reflect or measure disadvantage. These variables include: low-income, low educational attainment, high unemployment and low skilled occupations.

The dependent variables for the study are listed below.

### Primary outcomes

• Health status; measured using the WOMAC Osteoarthritis Index for OA of the knee (WOMAC LK3.0). Also self-administered, the WOMAC assesses pain, stiffness and physical function [13] and can be completed in less than 5 minutes. Two major studies have shown WOMAC pain, stiffness and physical function subscales are valid and the questionnaire is reliable and sensitive enough to detect changes in health status following a variety of interventions [14,15]

• Quality of life; measured using the Short Form 36v1 (SF-36) questionnaire. This 36 item questionnaire is self administered, and can be completed in about 15 minutes [16]. Scores for 8 sub components reflecting both physical and mental status can be generated. Reliability and validity have been established in numerous studies [16,17].

### Secondary outcomes

**Figure 1:**
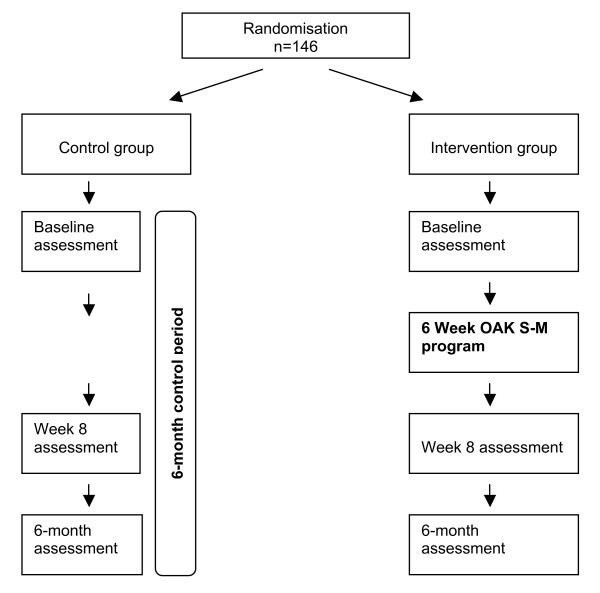
Study Design Flow Chart.

• Active range of motion of the knee joints; measured using a long armed Goniometer [18]. The reliability and validity of the goniometer to measure range of motion has been widely documented for knee flexion and extension [18,19].

• Strength of the hamstrings and quadriceps muscles will be measured using a Mecmesin Force Gauge Dynamometer. The dynamometer will be fixed via an adjustable arm to a portable steel frame and stool. Subjects will sit on the stool with hips and knees flexed to 90 degrees. Isometric strength in flexion and extension will be measured in this position. Each knee will be measured 3 times. The first measurement will be a practice and will be excluded from analysis. The two subsequent measures will be averaged for analysis.

• Pain will be assessed at weekly intervals from baseline to the 8-week follow-up assessment. (See Figure 1: Study Design Flow Chart). A 10 cm Visual Analog Scale (VAS) anchored at the left with "no pain" and at the right "worst pain imaginable" will be used for this assessment. The VAS is well established in clinical practice and research for measuring pain levels in arthritis populations [20].

• Functional mobility, using a Functional Knee Assessment Test (FKAT) will be assessed using a modification of the "Timed Up and Go" test (TUG). TUG is widely used to assess basic functional mobility in the elderly [21-23]. The test measures the time taken to stand from a chair and walk 6 m, turn around, return to the chair and sit down. For this study the addition of ascending and descending a 15 cm step will be added to the outward walk.

### Statistical Power Calculation

A priori power calculations for this study will be based on the quality of life outcome as measured by the SF-36. Sample size was calculated according to guidelines in the SF-36 Users Manual to determine differences in changes over time between the intervention and control groups using a repeated measures design allowing an inter temporal correlation between scores of 0.60 [16]. The pilot study SF-36 data showed an average improvement of 10 points across the eight domains measured. Assuming this level of improvement is achieved in the intervention group and there is no change in the control group and allowing for a 10% drop out rate, the number of participants required per group will be 60 [16]. In the pilot study, there was a drop out rate of 5% over 3 years, so allowing 10% is a conservative estimate. Differences in changes in functional ability measured using the WOMAC, similar in magnitude to those previously documented [24] would also be detectable in a sample of this size.

### Data Analysis

Data will be analyzed in a blinded manner. Treatment groups will be examined for comparability at baseline. Main comparisons between treatment groups will be performed using an intention to treat analysis. To test the effects of treatment, between group differences in changes over time will be examined using repeated measures ANOVA. Separate analysis will be conducted for each outcome variable. Statistical significance will be inferred at a 2-tailed p < 0.05. Results will not be adjusted for multiple comparisons as all outcomes of interest have been nominated a priori and such adjustment would likely render all findings of interest, despite their clinical importance, non-significant.

## Discussion

Self-management aims to motivate people to undertake the changes in behavior necessary to improve their lives. The preference of patients to actively manage their condition themselves is well matched to the aim of the multidisciplinary SM program to empower people to manage their condition [25]. People with OA of the knee have identified pain and problems with daily activities as the most important problems associated with their condition. Hence the results of the study will reflect their priorities. The outcome measures selected have demonstrated validity and are responsive within the range of change expected in response to the intervention.

This disease specific self-management program differs from other more generic arthritis programs. Using the skills and expertise of health professionals in a program providing disease specific education and self-management will provide a platform for behavior change that is not feasible with the limited knowledge base of lay leaders. The outcome measures are designed to reflect positive changes in pain, knee function and quality of life (intervention group), compared with those participants that are managed with conventional treatment (control group).

The study design compares the OAK program with the usual management of people with OA in the community. Randomisation to treatment or no treatment groups prevents conscious or unconscious bias [26]. Although blinding of group participants is not possible, the physiotherapists assessing outcome measures will be blinded to group allocation to minimize potential bias [27]. Using an intention to treat design will reflect the way the treatment will perform in the community and reduces the potential bias of drop out or non-compliant participants.

There is insufficient evidence to unequivocally claim that self-management is an effective treatment for osteoarthritis of the knee. The results of the study will provide evidence to guide clinicians and funding bodies to establish priorities regarding the provision of this disease specific program.

## Competing interests

The authors declare that they have no competing interests.

## Authors' contributions

SC and KB were responsible for writing the study protocol and drafting the manuscript. GC, CI, NC and JM assisted with study design and provided comments on the drafts and all authors approved the final version of the manuscript.

## Pre-publication history

The pre-publication history for this paper can be accessed here:



## References

[B1] Lorig K (2002). Partnership between expert patients and physicians. The Lancet.

[B2] Warsi A, Wang PS, LaValley MP, Avorn J, Solomon DH (2004). Self-management education programs in chronic disease. A systematic review and methodological critique of the literature. Archives of Internal Medicine.

[B3] Mullen PD, Laville EA, Biddle AK, Lorig K (1987). Efficacy of psychoeducational interviews on pain, depression, and disability in people with arthritis: MetaAnalysis. Journal of Rheumatology.

[B4] Superio-Cabuslay E, Ward MM, Lorig KR (1996). Patient education interventions in osteoarthritis and rheumatoid arthritis: a meta-analytic comparison with nonsteroidal antiinflammatory drug treatment. Arthritis Care and Research.

[B5] Lorig KR, Sobel D, Ritter P, Laurent D, Hobbs M (2001). Effect of a self-management program on patients with chronic disease. Effective Clinical Practice.

[B6] Lorig K, Mazonson P, Holman H (1993). Evidence suggesting that health education for self-management in patients with chronic arthritis has sustained health benefits while reducing health care costs. Arthritis and Rheumatism.

[B7] Newman S, Steed L, Mulligan K (2004). Self-management interventions for chronic illness. The Lancet.

[B8] Ofman JJ, Badamgarav E, Henning JM, Knight K, Gano AJ, Levan RK, Gur-Arie S, Richards MS, Hasselblad V, Weingarten SR (2004). Does disease management improve clinical and economic outcomes in patients with chronic diseases? A systematic review. The American Journal of Medicine.

[B9] Coleman S, Briffa K, Conroy H, Prince R, Carroll G, McQuade J (2008). Short and medium-term effects of an education self-management program for individuals with osteoarthritis of the knee, designed and delivered by health professionals: a quality assurance study. BMC Musculoskeletal Disorders.

[B10] Hootman JM, Sniezek JE, Helmick CG (2002). Women and arthritis: burden, impact and prevention programs. Journal of Women's Health & Gender-Based Medicine.

[B11] Bandura A (2005). The primacy of self-regulation in health promotion. Applied Psychology: An International Review.

[B12] Australian Bureau of Statistics (2001). Census of Population and Housing: Socio-Economic Indexes for Area's (SEIFA).

[B13] Bellamy N, Buchanan WW, Goldsmith CH, Campbell J, Stitt LW (1988). Validation study of WOMAC: a health status instrument for measuring clinically important patient relevant outcomes to antirheumatic drug therapy in patients with osteoarthritis of the hip or knee. Journal of Rheumatology.

[B14] Bellamy N (2002). WOMAC osteoarthritis index: User guide.

[B15] Bellamy N (2005). The WOMAC knee and hip osteoarthritis indices: development, validation, globalization and influence on the development of the AUSCAN hand osteoarthritis indices. Clinical and Experimental Rheumatology.

[B16] Ware JE, Kosinski MA, Gandek B (2002). SF-36 Health Survey Manual & Interpretation Guide.

[B17] Kantz ME, Harris WJ, Levitsky K, Ware JE, Davies AR (1992). Methods for assessing condition specific and generic functional status outcomes after total knee replacement. Medical Care.

[B18] Gogia P, Braatz JH, Rose SJ, Norton BJ (1987). Reliability and validity of goniometric measurements of the knee. Physical Therapy.

[B19] Watkins MA, Riddle DL, Lamb RL, Personius WJ (1991). Reliability of goniometric measurements and visual estimates of knee range of motion obtained in a clinical setting. Physical Therapy.

[B20] Creamer PL-C, Hochberg M, M C (1999). Determinants of pain severity in knee osteoarthritis: effect of demographic and psychosocial variables using 3 pain measures. Journal of Rheumatology.

[B21] Podsiadlo D, Richardson S (1991). The timed "Up and Go": a test of basic functional mobility for frail elderly persons. Journal of American Geriatric Society.

[B22] Vellas B, Wayne S, Romero L, Baumgartner R, Rubenstein L, Garry P (1997). One-legged balance is an important predictor of injurious falls in older persons. Journal of American Geriatric Society.

[B23] Huxham F, Goldie P, Patla A (2001). Theoretical considerations in balance assessment. Australian Journal of Physiotherapy.

[B24] Fransen M, Crosbie J, Edmonds J (2001). Physical therapy is effective for patients with osteoarthritis of the knee: a randomised controlled clinical trial. Journal of Rheumatology.

[B25] Walker-Bone K, Javaid K, Arden N, Cooper C (2000). Medical management of osteoarthritis. British Medical Journal.

[B26] Altman D, Bland J (1999). Treatment allocation in controlled trials: why randomise?. British Medical Journal.

[B27] Day S, Altman D (2000). Blinding in clinical trials and other studies. British Medical Journal.

